# *Neisseria sicca* Endocarditis Complicated by Intracranial and Popliteal Aneurysms in a Patient with a Bicuspid Aortic Valve

**DOI:** 10.1155/2013/895138

**Published:** 2013-02-05

**Authors:** Guillaume Debellemanière, Catherine Chirouze, Laurent Hustache-Mathieu, Damien Fournier, Alessandra Biondi, Bruno Hoen

**Affiliations:** ^1^Department of Infectious Diseases, University Medical Center of Besançon, 25030 Besançon, France; ^2^Department of Bacteriology, University Medical Center of Besançon, 25030 Besançon, France; ^3^Department of Neuroradiology, University Medical Center of Besançon, 25030 Besançon, France

## Abstract

We report a case of infective endocarditis due to *Neisseria sicca* complicated by intracranial and popliteal aneurysms and hepatic and splenic infarcts in a patient with a bicuspid aortic valve. No predisposing factor other than poor dental condition was found. The patient fully recovered after antibiotic therapy, aortic and mitral valve replacement, endovascular occlusion of the middle-cerebral artery aneurysm, and surgical treatment of the popliteal artery aneurysm.

## 1. Background

The *Neisseria* genus includes a wide range of species, *N. meningitidis* and *N. gonorrhoeae* being the two most frequently involved in infections in humans. Other species, which are part of normal oropharyngeal flora, are often referred to as “nonpathogenic *Neisseria*” although they may be responsible for serious conditions such as endocarditis, meningitis, osteomyelitis, vertebral osteomyelitis, and pneumonia. We report the case of a patient with *Neisseria sicca* endocarditis, complicated with mycotic aneurysms of the middle cerebral and popliteal arteries.

## 2. Case Presentation

A 41-year-old male patient with a known history of bicuspid aortic valve was admitted to hospital for persisting fever and flu-like syndrome. He had smoked 40 cigarettes a day for 10 years and denied any illicit drug use. Twenty days prior to admission, he had developed myalgia, anorexia, intermittent fever and fatigue. He had been evaluated by his general practitioner whose findings were unremarkable. One week later, laboratory test results were as follows: whole white blood cell count 16.7 G/L and C-reactive protein 336 mg/L. Urine culture was positive for *E. coli* (10³ CFU/ml) and the patient was given amoxicillin/clavulanate. Because his symptoms did not abate he was admitted to hospital.

On admission, temperature was 38.5°C, heart rate was 98 bpm, and blood pressure was 100/80 mmHg. Cardiac examination revealed a systolic murmur, maximal at the aortic area. Breath sounds were normal. The liver was moderately enlarged. There was no pedal edema. Neurological examination was unremarkable. Dental condition was poor.

The WBC count was 19.4 G/L.Serum ALT and AST levels were 184 U/L and 216 U/L, respectively;  *γ*-GT was 146 U/L; alkaline phosphatase was 133 U/L and C-reactive protein was 278 mg/L. Brain natriuretic peptide level was 217 pg/mL (0–100 pg/mL). Chest X-ray was normal. Electrocardiogram showed a sinus rhythm and a heart rate of 100 bpm.

Transesophageal echocardiography showed a bicuspid aortic valve with a 16 × 22 mm vegetation attached on the ventricular leaflet, associated with a grade 2 aortic regurgitation and an 8 mm aortic ring abscess. After blood cultures were drawn, antibiotic therapy with amoxicillin/clavulanate and gentamicin was initiated. Four sets (4 aerobic and 2 anaerobic) of blood cultures grew gram-negative diplococci within 24 hours. Treatment was then switched to ceftriaxone and gentamicin. After 24 h of incubation aerobically, the organism was identified as *Neisseria sicca*, using the biochemical identification gallery (API strip 4H, BioRad). This identification was subsequently confirmed after 16S rRNA gene sequencing (100% homology with *Neisseria sicca *ATCC 29256). Gentamicin was switched to ofloxacin at this time, after four days of antibiotic therapy.

**Figure 1:**
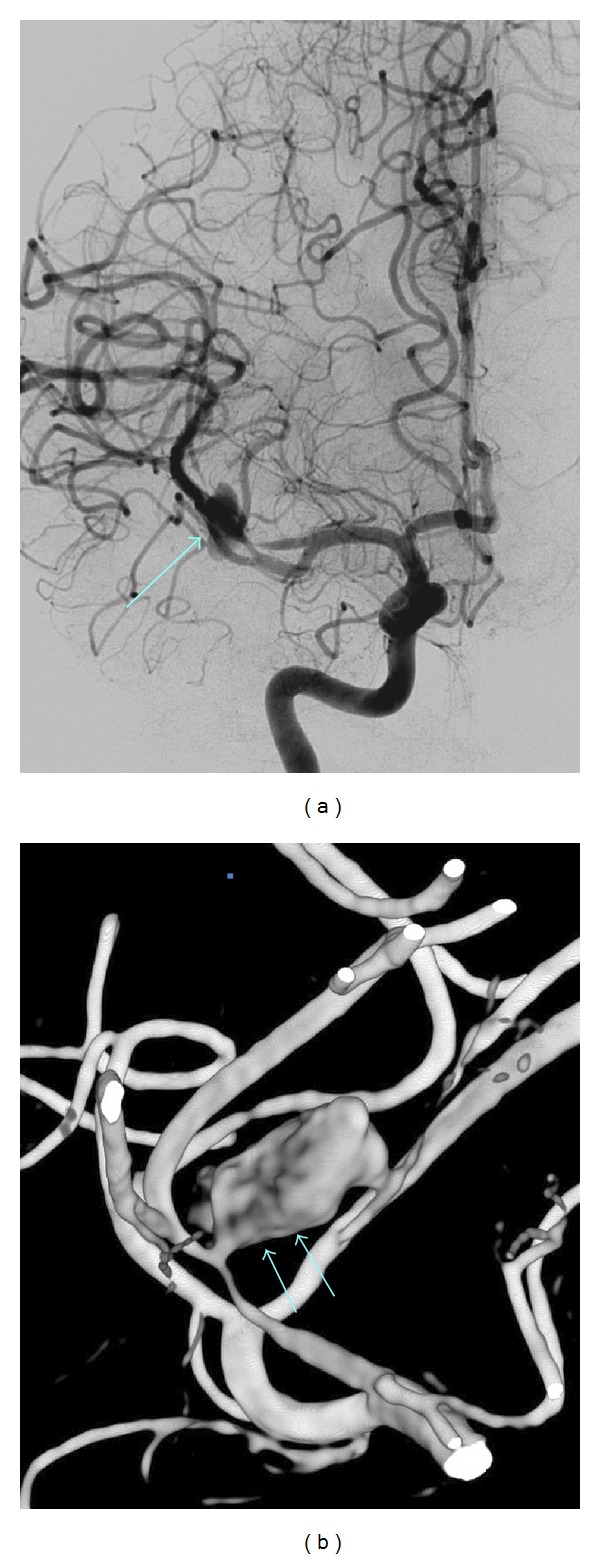
Septic aneurysm of the superior branch of the right middle cerebral artery bifurcation. Anteroposterior view of the right internal carotid angiogram showing the aneurysm (arrow) and visualization of the aneurysm on three-dimensional reconstruction (double arrows).

On the fifth day of hospitalization, the patient developed congestive heart failure and was transferred to the cardiac intensive care unit. A new transesophageal echography showed an enlarged aortic vegetation, an extension of the aortic ring abscess, and a new 4 mm mitral vegetation on the anterior leaflet. CAT scan of the thorax, abdomen, and pelvis showed multiple infarcts in the spleen, liver, and kidneys. Brain MRI revealed multiple areas of embolic infarcts. Ciprofloxacin was substituted for gentamicin. Urgent valve surgery was decided and performed by Day 7. Operative findings included massively calcified bicuspid aortic valve with endocarditis lesions, abscess of the aortic ring, and a vegetation of the anterior mitral leaflet. Both aortic and mitral valves were replaced with St. Jude Medical mechanical prosthetic valves. Cardiac valve cultures were sterile. Postoperatively, the patient developed an acute atrioventricular block that necessitated the implantation of a cardiac pacemaker. On Day 16 (9 days pos-top), the patient developed acute pain in his right calf, which was related to a 2 × 3 cm pseudoaneurysm of the popliteal artery. Indication for surgery was postponed. On Day 35, the patient remained afebrile, neurological examination was normal, calf pain was moderate at rest, but walking was impossible. C-reactive protein was 59 mg/L. Antibiotic therapy was simplified to oral ciprofloxacin monotherapy. Repeat CAT scan evaluation showed complete (liver, kidney) and partial (spleen) resolution of infarcts and evidenced a new cerebral lesion corresponding to an aneurysm on the upper branch of the right middle cerebral artery, measuring 10 mm in its largest diameter, which was confirmed by angiography (Figure 1). At the same time, arteriography of the right leg showed that the diameter of the popliteal aneurysm had increased to 5 cm.

**Figure 2:**
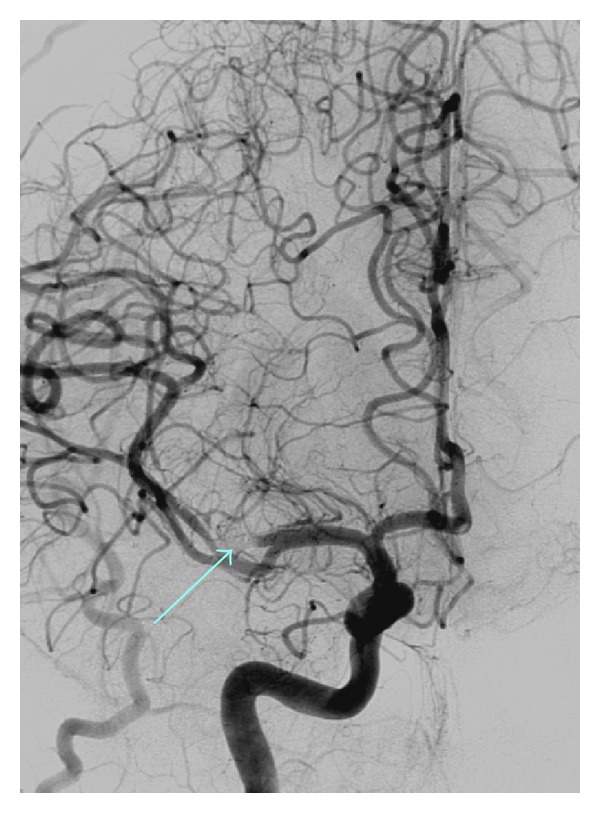
Anteroposterior view of the right internal carotid angiogram after endovascular treatment showing occlusion of the parent artery by coils (arrow) and complete exclusion of the aneurysm.

It was decided to perform first endovascular treatment of the intracranial aneurysm by parent artery occlusion. Balloon test occlusion of the superior branch of the middle cerebral artery showed retrograde circulation from collateral leptomeningeal branches without any clinical changes. Successful endovascular occlusion of the parent artery was performed using coils (Figure 2). Postoperative course was uneventful.

Two days later, the popliteal aneurysm was treated surgically, with a venous graft, with no complication.

Antibiotic therapy was stopped after 67 days and the patient was discharged by Day 68. Dental care was performed a few days after discharge. He made full recovery and no relapse had occurred 6 months after discharge. 

## 3. Discussion

*Neisseria sicca* is a rare cause of endocarditis. To the best of our knowledge, only twenty-one definite cases, including this one, have been published since 1918. Sixteen cases have been described in the antibiotic era in French, English, or Spanish language [1–13]. Predisposing conditions were found frequently, which included intravenous drug use in 6 cases [4, 7, 9–11], prior native valve disease in 4 cases ([5, 9], present case), and prosthetic valve in 1 case [3]. Endocarditis was located on the tricuspid valve in 4 of the 5 intravenous drug users, and was complicated with pulmonary embolisms in all of them [7, 9, 11]. Cerebral embolisms or haemorrhage were reported in 6 cases ([2–4, 6, 9], present case), with a fatal issue in one case [4]. Aneurysms of sinus of Valsalva [5] and calf [6, present case] were described.

## 4. Conclusion

It appears that infectious endocarditis due to *Neisseria sicca* or other so-called non-pathogenic *Neisseria* are frequently complicated by systemic embolisms and/or aneurysms. These aspects should be known to clinicians who care for *Neisseria* endocarditis.
